# The problem with petrous? A consideration of the potential biases in the utilization of *pars petrosa* for ancient DNA analysis

**DOI:** 10.1080/00438243.2019.1694062

**Published:** 2020-01-10

**Authors:** Sophy Charlton, Thomas Booth, Ian Barnes

**Affiliations:** a Department of Earth Sciences, Natural History Museum, London, UK; b PalaeoBARN, School of Archaeology, University of Oxford, Oxford, UK; c Ancient Genomics Laboratory, The Francis Crick Institute, London, UK

**Keywords:** Ancient DNA, petrous, bias, palaeogenomic data

## Abstract

Advances in NGS sequencing technologies, improved laboratory protocols and new bioinformatic workflows have seen huge increases in ancient DNA (aDNA) research on archaeological materials. A large proportion of aDNA work now utilizes the petrous portion of the temporal bone (*pars petrosa)*, which is recognized as an excellent skeletal element for long-term ancient endogenous (host) DNA survival. This has been significant due to the often low endogenous content of other skeletal elements, meaning that large amounts of sequencing are frequently required to obtain sufficient genetic coverage. However, exclusive sampling of the petrous for aDNA analysis introduces a new set of potential biases into our scientific studies – and these issues are yet to be considered by ancient DNA researchers. This paper aims to outline the possible biases of utilizing petrous bones to undertake aDNA analyses and highlight how these complications may potentially be overcome in future research.

## Introduction

The past 10 years have seen huge progress in the field of ancient DNA (aDNA) analysis, allowing whole genomes and new genetic data to be generated from a wide range of archaeological materials, from ever increasing temporal and geographical ranges. There is now a growing awareness of the utility of ancient DNA studies in obtaining archaeologically informative information on past population histories, as well as determining processes such as local population extinction, expansion, migration, admixture, population replacement, and changes in effective population size, in both humans and fauna (e.g. Olalde et al. 2018, 2019; Brace et al. 2019; Fu et al. 2016; Frantz et al. 2016; Daly et al. 2018; Barlow et al. 2018; Palkopoulou et al. 2015; Verdugo et al. 2019). To a large extent, these advances have been facilitated by significant methodological developments, most notably in next generation sequencing (NGS) technologies, and also in laboratory methods allowing for more efficient recovery of ultra-short DNA fragments (e.g. Dabney et al. 2013) and improved library preparation techniques (e.g. Meyer and Kircher 2010; Rohland et al. 2015; Carøe et al. 2018). These methodological advancements have allowed for increased success in the recovery and sequencing of ancient DNA, and have significantly improved the utility of the data generated, even from material of significant age or from more temperate environments (e.g. Meyer et al. 2012; Fu et al. 2014; Hajdinjak et al. 2018; Lazaridis et al. 2016; Skoglund et al. 2016).

However, whilst NGS technologies have allowed ancient DNA research to provide much greater insights than earlier polymerase chain reaction (PCR) and Sanger sequencing-based methods, some alternative problems are encountered when applying NGS methods to archaeological material. One particular issue is that NGS sequencing of archaeological material often utilizes a shotgun approach, meaning all DNA contained within the sample is sequenced, rather than a targeted approach with a focus on specific sequences. The highly degraded nature of DNA recovered from archaeological and museum materials has long been well appreciated, and is in essence a defining characteristic of the field (Díez-Del-Molino et al. 2018, Box 2). However, older PCR-based approaches failed to characterize that a significant proportion of the DNA recovered from archaeological remains appears to derive from soil bacteria, as well as other contaminants, or in some cases, is unidentifiable. DNA which can be identified as deriving from the host organism (the ‘endogenous content’), often makes up a very small amount of the total DNA yield; in many instances, less than 1%, meaning that large amounts of sequencing are often required to obtain sufficient genome coverage to draw meaningful inferences.

One solution to this problem has been the recognition that the petrous portion of the temporal bone (*pars petrosa*; from here on in referred to as ‘the petrous’) provides the highest endogenous (host) DNA preservation of any skeletal element (Gamba et al. 2014; Pinhasi et al. 2015). The petrous is located at the base of the crania between the sphenoid and occipital bones, and houses the vestibulo-cochlear organs which are responsible for hearing and balance. The petrous is known to be very robust, and has been found to exhibit unique microstructural characteristics and a lack of bone remodelling throughout life (Kontopoulos et al. 2019), which may contribute to enhanced aDNA survival. In particular, the otic capsule (also known as the osseous labyrinth or bony labyrinth), which surrounds the vestibulo-cochlear organs of the inner ear and includes the cochlea, vestibule and three semi-circular canals, is known to be one of the densest osseous tissues in the human body (Harvig et al. 2014), and as such, has been the preferred target for sampling for aDNA analyses. Endogenous DNA yields from the otic capsule can be up to 180-fold higher than those obtained from other skeletal elements within the same individual (Gamba et al. 2014; Pinhasi et al. 2015). Targeted sampling of the petrous has therefore now been widely adopted within many recent aDNA studies on archaeological skeletal material (e.g. Gallego Llorente et al. 2015; Moreno-Mayar et al. 2018; Brace et al. 2019).

## The problem with petrous

Whilst the utilization of petrous bones in ancient DNA studies has resulted in increased yields of endogenous aDNA from archaeological skeletal material from a range of geographical locations and time periods, the increased, and in some instances, wholesale inclusion of petrous as a substrate for aDNA analysis has however led to a number of problems. These issues we feel have not yet been considered by the ancient DNA community, but it is imperative they are addressed, due to the increasing number of aDNA publications each year which utilize archaeological skeletal material.

Firstly, the adoption of petrous as the preferred skeletal substrate for ancient genetic analyses has unsurprisingly led to increased destruction of petrous bones and crania. This is problematic as there are only two petrous within the crania, and it may often be the case that only one may be preserved, particularly in prehistoric or disarticulated assemblages. If sampling results in the removal of a significant portion of the petrous, then osteological information may also be lost, and if the entire otic capsule is utilized for aDNA extraction, then future analyses cannot be undertaken on the same specimen (Hansen et al. 2017). Furthermore, the petrous also has a range of applications for scientific analysis beyond ancient DNA research. For example, it has been suggested to be morphologically informative, not only in *Homo sapiens* but also other hominin species (Spoor et al. 2003; de León et al. 2018), and as the otic capsule does not undergo any further remodelling after the age of 2 years, it has utility in determining both early childhood diet (Jørkov et al. 2009) and childhood origins and mobility (Harvig et al. 2014). Total destruction of the petrous therefore means that this additional information is lost. Although a more minimally destructive sampling technique for petrous has now been published (e.g. Sirak et al. 2018), along with methods for CT scanning of specimens prior to sampling (Alberti et al. 2018), the petrous remains a limited resource – even more so than, for example, teeth or cortical bone, of which there may be multiple sampling locations per individual.

The ethical considerations around the destructive sampling of human remains for biomolecular analyses have been variously discussed in recent years (Walker 2000; Austin et al. 2019), and there is now also an increased awareness of the ethical considerations surrounding the growing demand for samples for ancient DNA analysis and the increasing number of studies utilizing palaeogenomic data (Redfern and Clegg 2017; Holst 2017; Elliott 2009; Kaestle and Ann Horsburgh 2002; Makarewicz, Marom, and Bar-Oz 2017; Prendergast and Sawchuk 2018; Bardill et al. 2018). A full exploration of the ethical considerations surrounding the utilization of petrous for aDNA analyses is beyond the scope of this paper, but we believe that ethical guidelines need to be more fully extended to the sampling of petrous bones in future, considering aspects such as the methodological approach proposed for aDNA analysis.

The second issue arising, and that which is the main focus of this paper, is that it is possible that the adoption of a sampling methodology solely focused around petrous bones may be introducing a new set of biases into our scientific studies. This is because in mortuary or funerary contexts, skulls are frequently treated differently to the post-cranial skeleton – and this is a phenomenon which can be seen across multiple temporal and geographical settings throughout the archaeological record.

In varied archaeological contexts, we see crania buried or deposited in different ways to the post-cranial skeleton. There are many instances in which skulls are found to be buried without the post-cranial elements, sometimes as caches of crania, or as ‘skull nests’. One such example is from the Mesolithic site of Ofnet in Bavaria, Germany, where two ‘nests’ (containing 27 and six skulls, respectively) were recovered. The presence of cervical vertebrae and cut-marks has been interpreted as suggesting decapitation of fleshed remains (Schulting 1998). Crania are also found variously deposited in pits, postholes or foundation deposits of (residential) structures at a wide range of different sites globally, and from different chronological time periods, such as, for example, the Mississippian culture complex site of Moundville in Alabama, USA (Knight and Steponaitis 1998), at a range of Scottish Iron Age sites (Armit and Ginn 2007), at a number of Levantine Middle Pre-pottery Neolithic B period sites such as ‘Ain Ghazal and Jericho (Kuijt 2008), and from various Romano-British sites (Marsh and West 1986; Mays and Steele 1996). Skulls are also often purposively deposited in other unusual locations. At the Mesolithic site of Kanaljorden in Motala, Sweden, disarticulated crania from at least 10 individuals were recovered from a large stone structure constructed within a shallow lake, at least two of which had been mounted on wooden stakes (Gummesson, Hallgren, and Kjellström 2018; Hallgren 2011). In a number of different time periods and geographical locales, we also see evidence of ‘skull cults’ or ‘cults of the head’. The reasons behind these skull cults are likely varied and diverse, but in some instances may have been tied to practices of head-hunting and trophy skulls, whereas in others may have been a product of funerary practice or linked to head veneration. The European Iron Age ‘cult of the head’ has been widely explored (Armit 2012), as have the skull cults of the Near Eastern Pre-Pottery Neolithic (Testart 2008; Kuijt 2008; Kuijt, Özdoğan, and Parker Pearson 2009). Furthermore, in some instances, we see crania being modified post-mortem and also utilized by living populations, for example as skull cups. This practice is seen in a variety of archaeological contexts, for example, at the Magdalenian site of Gough’s Cave, Somerset, UK (Bello, Parfitt, and Stringer 2011), the Neolithic site of Herxheim, Germany (Orschiedt and Haidle 2006), the Bronze Age site of El Mirador Cave, Sierra de Atapuerca, Spain (Cáceres, Lozano, and Saladié 2007), and the Huarpa-era (400–700 AD) site of Nawinpukio, Peru (Finucane 2008).

With disarticulated or commingled material, there are additional considerations with regards to skulls, however, as there is no guarantee that the cranial and post-cranial remains within the assemblage belong to the same individuals. Indeed, some individuals may only be represented by either cranial or postcranial elements, respectively. Linked to this, at some sites with disarticulated remains or evidence of secondary mortuary rites, we see either an over- or under-representation of crania. For example, skulls are under-represented at West Kennet, a Neolithic chambered tomb in Wiltshire, UK, but are over-represented at the nearby causewayed enclosure site of Windmill Hill (Parker Pearson 2003, 50). In some cases, even seemingly articulated skeletons may be biased in the same way, however. For example, in some instances skeletons may be composite – i.e. composed of skeletal elements from multiple individuals. At the site of Cladh Hallan, in the Scottish Outer Hebrides, for example, two burials discovered as foundation deposits buried under Bronze Age houses were in fact composites of five individuals, with the crania in both burials and one mandible being replaced by substitutes from other individuals (Kuijt, Özdoğan, and Parker Pearson 2009). There are also many instances of skulls being removed from articulated inhumations or additional skulls being added into burials, from the Palaeolithic (e.g. Kebara 2 adult male from Kebara Cave, Israel (Bar-Yosef et al. 1992)) onwards.

Quite why skulls are frequently treated differently to the post-cranial skeleton in mortuary contexts is unclear. In some instances, the skull of the deceased may have been viewed as the most representative element of that individual, and as embodying them – with the post-cranial skeleton being of lesser importance perhaps – and may have served to emphasize ancestral beliefs, identity, community, or ties to a specific place (Parker Pearson 2003, 161). Others have suggested that the head is of significant importance as it may have been viewed as a receptacle for the spirit or power of the deceased individual (Schulting 1998). Conversely, some have proposed that removal of skulls may have separated the crania from the physical characteristics of the deceased, serving to homogenize the past and act as a levelling mechanism in death, whilst still allowing the crafting of memory (Kuijt 2008).

Removing crania, either peri- or post-mortem, and/or separate selective burial of crania in alternate locations, can therefore be seen as a recurring theme within the global archaeological mortuary record, and raises interesting ideas about deliberate manipulation, disarticulation, and curation of human remains in the past. It is also frequently skulls which appear to have been curated and preferentially chosen for collective burial, rather than other skeletal elements. This therefore raises interesting considerations surrounding the differential treatment of the head, and whether having a sampling strategy which is only focused around petrous or crania may be introducing additional biases to the analysed assemblage, and may mean that the sample selection is not representative of the death population as a whole.

Overall therefore, it can be seen therefore that the adoption of petrous as the preferred substrate for aDNA analyses raises a number of important issues; notably, the increased destruction of a limited resource, and the potential biases introduced to scientific studies by the sampling of solely crania.

## The case study of Aveline’s hole

Recent work undertaken on the human skeletal material from the site of Aveline’s Hole provides a useful case study for the potential biases and problems of utilizing only petrous samples for genetic analyses. Aveline’s Hole, Burrington Combe, Somerset, is Britain’s earliest cemetery site, dating to the Early Mesolithic, c. 8300 cal. BC (Schulting 2005). Whilst a significant dating programme and dietary isotopic analyses had previously been undertaken on the human skeletal material from the site (Tratman 1977; Hedges et al. 1987; Schulting 2005), aDNA analysis was only recently undertaken on the remains (Brace et al. 2019). Previously, 23 radiocarbon dates had been carried out on human skeletal remains from Aveline’s Hole, predominantly on ulnae and humeri, although one cranium was also dated (Tratman 1977; Schulting 2005). All radiocarbon determinations placed the human remains within the Early Mesolithic, in the late ninth millennium BC, and it was suggested that all dated skeletons belonged to a single phase of burial activity at the site, spanning less than 200 years (Schulting 2005). This radiocarbon dating established a narrative that the site was purely a Mesolithic cemetery site, with no evidence for subsequent burial activity, and which had remained ‘sealed’ until its discovery in 1797 and subsequent excavation (Tratman 1977; Schulting 2005).

As part of a larger project exploring genetic diversity in British prehistory, 13 human skeletal samples were analysed for aDNA from the Aveline’s Hole assemblage. Of these, seven showed endogenous content close to zero, and a further four had endogenous values of 1–3%, thereby making them unsuitable for whole genome sequencing (Brace et al. 2019; Schulting et al. 2019). However, two samples did show good endogenous preservation (68% and 14%, respectively), and were therefore chosen for additional sequencing and analysis. Both of these samples were petrous bones (Brace et al. 2019; Schulting et al. 2019).

Surprisingly, when analysed, both petrous displayed ancestry akin to the genetic cluster termed ‘Aegean Neolithic Farmers’ (ANF), consistent with European Neolithic farmer ancestry. However, as discussed above, previous radiocarbon dating had placed the site of Aveline’s Hole firmly within the Early Mesolithic of Britain, in the late ninth millennium BC, a point at which there is not yet evidence of ANF ancestry within mainland European populations (Mathieson et al. 2015; Lazaridis et al. 2016). Consequent additional AMS dating of the Aveline’s Hole petrouses has however revealed that they instead belong to the Early Neolithic of Britain, c. 3750–3470 cal. BC (Schulting et al. 2019), when high levels of ANF ancestry would be anticipated (Brace et al. 2019).

Subsequently, it was also possible to obtain data from two post-cranial elements (a femur and a tibia) from Aveline’s Hole, using a reduced representation method that recovers a targeted set of sites from across the genome. Although the resulting dataset was smaller, both of these samples displayed genetic signatures indicative of Western Hunter-Gatherers (WHG), as would be anticipated from a British Early Mesolithic site. The WHG genetic cluster is seen across Europe from c.12500 BC, and is genetically very distinct from the later ANF cluster which accompanies the arrival of farming in Europe – suggesting that the agricultural transition was driven by migration rather than acculturation (Fu et al. 2016; Haak et al. 2015; Gamba et al. 2014; Skoglund and Mathieson 2018; Lazaridis et al. 2014; Brace et al. 2019).

It therefore appears that the cranial and post-cranial remains at Aveline’s Hole have divergent depositional histories, are from different time periods, and relate to completely different populations. In this instance, we see a hiatus of nearly five millennia in the deposition of human remains at Aveline’s Hole – and it is only due to previous and additional subsequent radiocarbon dating programmes that this anomaly between the cranial and post-cranial remains in the assemblage was fully recognized and understood. This combined biomolecular approach was therefore crucial in the interpretation of the site and its depositional history. Furthermore, it also highlights that whilst in the case of the Aveline’s Hole skeletal material, sampling of the petrous for aDNA analysis helped to provide a better understanding of the site’s use and mortuary history, this was greatly assisted by previous study of the material and other biomolecular analyses. In the analysis of less well-studied material, however, the reverse may in fact occur, in that sampling of solely petrous for aDNA would provide an unrepresentative or biased sample, which may not be recognized, thereby resulting in a biased or incomplete interpretation of the site and the human skeletal assemblage. It is not expected that similar analyses at other archaeological sites will be as unique, extreme or unexpected as at Aveline’s Hole, but the site serves as a cautionary tale in the utilization of solely petrous material to obtain genetic information and make population history inferences.

A reanalysis of the Aveline’s Hole assemblage, considering the new genetic data, AMS dates and also isotopic data, and the newly discovered multiple phases of deposition of human remains within the cave both in the Early Mesolithic and Early Neolithic periods, can be found within Schulting et al. (2019).

## Suggestions and considerations for future work

Given the issues outlined above, we propose a number of suggestions and considerations for future work. Primarily, these revolve around the need for greater consideration to be given to sampling procedures used and the selection of skeletal elements for aDNA and other biomolecular analyses. There are also a number of potential ethical issues surrounding the sampling of petrous bones, which require careful consideration, but of which a full exploration is beyond the scope of this paper.

In terms of future sample selection, whilst we recognize that it is preferable in many cases to sample the same skeletal element of all individuals within a study to avoid duplication of individuals, recognition of the biases of this approach need to be acknowledged, especially when dealing with disarticulated and prehistoric material. In particular, the sole use of petrous material for ancient DNA studies can, as the Aveline’s Hole case study shows, result in significant bias in the genetic data obtained, and potential misinterpretation of sites or assemblages. Furthermore, given that crania often appear to be given differential treatment in mortuary contexts globally, we must acknowledge that skulls may represent a biased proportion of both the living and the death assemblage. It may be the case that in certain archaeological contexts, crania may only be representative of a certain subset of the population – be this in terms of social status, sex, ethnicity, age or kinship. It is also important to note that the biases outlined here for petrous material can therefore equally be applied to all other cranial elements sampled for aDNA analysis – for example, teeth are often also frequently sampled within palaeogenetic studies as they too have been demonstrated to be a good source of endogenous aDNA (Damgaard et al. 2015).

A recent paper has also suggested the sampling of auditory ossicles (the malleus, incus and stapes) may provide an alternative to the petrous for ancient DNA analyses, as they provide comparable endogenous DNA yields and levels of complexity (Sirak et al. 2019). However, ossicles are subject to the same biases as discussed above in regards to petrous bones, particularly given that they too form part of the crania. Furthermore, ossicles can also be morphologically informative (Quam and Rak 2008; Quam et al. 2013; Stoessel et al. 2016), and importantly, are only occasionally preserved within archaeological collections due to their small size and tendency to become loose post-mortem.

In cases where it is thought that an additional bias may be introduced into the analysis by only sampling crania (be this petrous, auditory ossicles or teeth) – perhaps particularly when analysing prehistoric, disarticulated or commingled skeletal remains, or at sites where there is an over- or under-representation of crania – it may be useful or preferable to additionally sample postcranial elements. Recently, the outermost layer of long bones has been shown to also be a good substrate for ancient DNA research, despite being routinely removed prior to DNA extraction (Alberti et al. 2018). Targeted sampling of the outermost bone layer can result in up to a 50-fold increase in endogenous DNA content when compared to that sampled from trabecular bone (Alberti et al. 2018). It is also important to note, however, that many of the same issues raised here with regard to sample selection for ancient DNA studies may also be applicable to other biomolecular techniques (e.g. stable isotope analyses, radiocarbon dating).

Finally, linked to the above points, is the recognition that the biases of single element selection (be this petrous, other cranial elements, or otherwise) as a sampling methodology must also now be more closely considered in the submission of destructive sampling requests for skeletal material. As destructive sampling requests for petrous material increase, institutions, universities, museums or archaeological contractors holding skeletal material face increased pressures in determining whether applications should be approved, and in calculating the balance between gain in scientific and archaeological knowledge against the loss of material. The scientific utility of archaeological and anthropological collections is widely recognized, and those curating or in charge of such collections occupy a unique position in promoting long-term preservation and protection of skeletal material whilst also providing access and materials to researchers (Walker 2000; Sholts, Bell, and Rick 2016; Austin et al. 2019). Whilst most museums and institutions now have policies for destructive sampling requests and committees which assess proposals submitted, these policies are not always substrate specific. Greater dialogue between the different stakeholders associated with skeletal remains collections is now needed, as well as potentially biomolecular technique (e.g. aDNA analysis, proteomics, AMS dating, isotopic analyses) and substrate (e.g. petrous, post-cranial remains, teeth, dental calculus, hair, tissue) specific destructive sampling policies. A full exploration of the ethical considerations surrounding destructive sampling is sadly beyond the scope of this paper but is something that also needs to be explored more closely alongside potential biases in sample selection, particularly in relation to petrous bones.

## Conclusions

It is important to note that the authors here do not advocate for requests for the sampling of petrous for aDNA analysis to be discontinued or for the wholesale cessation of petrous use in aDNA studies – particularly given the hugely informative genetic information that aDNA studies can provide, and the knowledge that the petrous provides the highest endogenous preservation of any skeletal element. However, with increasing numbers of applications, and the finite number of petrous bones available for study within UK collections, the methodological approaches proposed and potential outcomes of aDNA analyses on skeletal material must be weighed up by all those involved in the curation, analysis and research of skeletal remains.

The Aveline’s Hole case study provides a clear example of the potential biases which may arise when utilizing a single skeletal element, and specifically petrous bones, for genetic analyses, and on broader scale, of the potential biases that may be incurred when choosing samples for any kind of scientific analysis. The Aveline’s Hole case study also highlights the need for combined biomolecular approaches to skeletal material. The inclusion of other biomolecular techniques such as AMS dating and stable isotope analyses alongside genetic studies will help to mitigate some of these biases, as well as increasing our understanding of the archaeological past.

We hope that the discussions and considerations outlined within this paper may of use or interest to all those involved in both the sampling and analysis of skeletal material for ancient DNA analysis, as well as all those involved in the curation of skeletal material in museums, universities, public institutions and archaeological contractors. The increasing number of researchers and laboratories undertaking aDNA research means that destructive sampling requests are only set to increase. Whilst these genetic studies can provide us with new insights into the past, and improve our understanding of past population histories, we must now acknowledge the potential biases and issues that may be incurred in sample selection approaches, what this may mean for the data which we generate, and how this may affect our interpretations of the archaeological past.
